# The effect of renal function on the clinical outcomes and management of patients hospitalized with hyperglycemic crises

**DOI:** 10.3389/fendo.2024.1445040

**Published:** 2025-01-09

**Authors:** Sumaya N. Almohareb, Norah Aljammaz, Nada Yousif, Mayar Sunbul, Raghad Alsemary, Lama Alkhathran, Mohammed Aldhaeefi, Omar A. Almohammed, Abdulrahman I. Alshaya

**Affiliations:** ^1^ Department of Pharmacy Practice, College of Pharmacy, King Saud bin Abdulaziz University for Health Sciences, Riyadh, Saudi Arabia; ^2^ King Abdullah International Medical Research Center, Riyadh, Saudi Arabia; ^3^ Pharmaceutical Care Department, King Abdulaziz Medical City, Ministry of the National Guard-Health Affairs, Riyadh, Saudi Arabia; ^4^ Department of Clinical and Administrative Pharmacy Sciences, Howard University College of Pharmacy, Washington, DC, United States; ^5^ Department of Clinical Pharmacy, College of Pharmacy, King Saud University, Riyadh, Saudi Arabia

**Keywords:** diabetic ketoacidosis, hyperosmolar hyperglycemic state, chronic kidney disease, insulin therapy, hypoglycemia, anion gap, DKA, HHS

## Abstract

**Background:**

The global prevalence of diabetes has been rising rapidly in recent years, leading to an increase in patients experiencing hyperglycemic crises like diabetic ketoacidosis (DKA) and hyperosmolar hyperglycemic state (HHS). Patients with impaired renal function experience a delay in insulin clearance, complicating the adjustment of insulin dosing and elevating hypoglycemia risk. Accordingly, this study aims to evaluate the impact of renal function on the safety and efficacy of insulin use in patients with isolated DKA or combined DKA/HHS.

**Methods:**

A retrospective observational study was conducted at King Abdulaziz Medical City, Saudi Arabia, from January 2016 to December 2021. Eligible patients were ≥18 years, had a confirmed diagnosis of isolated DKA or combined DKA/HHS, presented with an anion gap (AG) of ≥ 16 mmol/L, and received insulin either via continuous infusion or as bolus doses. Patients were categorized into normal kidney function and patients with chronic kidney disease (CKD). The primary outcome was to determine the difference in time to close the AG between the two groups. Statistical analyses were performed using SAS® software.

**Results:**

Out of 319 screened patients, 183 patients met the inclusion criteria. The patients were divided into normal kidney function (43.2%) and CKD (56.8%) groups. The average eGFR for patients with normal kidney function was 93.7 ± 32.5 mL/min/1.73m^2^ compared to 33.4 ± 14.3 mL/min/1.73m^2^ for patients with CKD. The time to close AG was similar between patients in the normal kidney function and CKD groups (22.6 ± 16.0 hours vs. 24.5 ± 17.5 hours, *p*=0.4475). However, the patients’ length of stay in hospital (3.4 ± 2.5 days vs. 5.2 ± 4.0 days; *p*=0.0004) and ICU (2.5 ± 1.8 days vs. 4.0 ± 2.8 days; *p*=0.0453) were both significantly longer for patients with CKD. Hypoglycemic events were low in our study with only four documented cases among patients with CKD.

**Conclusion:**

This study provides insights into DKA management and outcomes in patients with normal and impaired renal function. The time required to close AG was comparable between the two groups. Larger, multi-center studies are needed to validate these findings and explore additional factors that may impact the management of DKA in patients with CKD.

## Introduction

The global prevalence of diabetes mellitus (DM) has been rising rapidly over the past years. In 2021, diabetes affected over half a billion of people worldwide and this number is expected to exceed 700 million in 2045 (1). The high incidence and prevalence of diabetes justify a rise in number of patients presenting to emergency departments with hyperglycemic crises, including diabetic ketoacidosis (DKA) and hyperosmolar hyperglycemic state (HHS) (2). DKA is characterized by a triad of hyperglycemia, ketonemia or ketonuria, and acidosis (2, 3). While HHS is distinguished by profound hyperglycemia and hyperosmolarity with no detectable ketones produced (4). Combined DKA/HHS features have been reported in some patients presented with hyperglycemic crises (5). Since these complications occur because of the reduction of the circulating insulin in the body, the mainstay of management includes insulin administration (2).

Normally insulin is metabolized via the kidneys into amino acids with an estimated renal clearance of 200 ml/min (6). As a result, patients with impaired renal function encounter a delay in insulin clearance, complicating the adjustment of insulin dosing, and increasing the risk of hypoglycemic events (3, 7). Additionally, patients with chronic kidney disease (CKD) commonly exhibit insulin resistance, which progressively worsens as the glomerular filtration rate (eGFR) declines (8, 9). Furthermore, renal dysfunction patients frequently develop metabolic acidosis, which is caused by a decrease in ammonium excretion and an increase in hydrogen ion retention, as well as a decrease in bicarbonate synthesis (10, 11). Hence, the variation of insulin kinetics in patients with CKD, in addition to the complications of the renal disease itself, is regarded as a challenge to practitioners when determining the appropriate insulin dosing (10, 11). Even though the pathophysiology of DKA and HHS differ in patients with renal disease, the treatment approach is currently similar to patients with normal renal function (12).

Numerous studies have attempted to investigate different dosing strategies in the management of hyperglycemic crises in patients with normal kidney function. However, there is a lack of studies addressing the management of DKA in patients with chronic kidney disease (13, 14). This necessitates the conduct of additional clinical studies to determine the proper treatment protocol for this group of patients. Therefore, the objective of our study is to evaluate the effect of renal function on the safety and efficacy of insulin use in the management of patients with isolated DKA or combined DKA/HHS.

## Methods

We performed a single-center, retrospective study at King Abdulaziz Medical City, a tertiary academic institution in Riyadh, Saudi Arabia. This study was approved by King Abdullah International Medical Research Center Institutional Review Board (SP20/435/R). Patients presented to emergency department with labeled diagnosis of hyperglycemic crises between January 1st, 2016, and December 31st, 2021, were identified using a hospital reporting system. Patients included if they were 18 years of age or older, had a confirmed diagnosis of DKA or combined DKA/HHS, presented with an anion gap (AG) of ≥ 16 mmol/L at admission, and received insulin either via continuous infusion for ≥ 1 hour or as bolus doses. Combined DKA/HHS was defined as confirmed diagnosis of HHS with high serum osmolality in addition to elevation of AG on presentation. Major exclusion criteria included patients undergoing any renal replacement therapy modality, isolated HHS, or incomplete electronic health records. Patients were categorized into normal kidney function group and CKD group. CKD was defined as an eGFR of < 60 ml/min for three months or more (15).

Baseline characteristics were collected at admission and included patient’s age, gender, weight, height, type of diabetes, creatinine clearance (CrCl), eGFR, serum osmolality, AG, bicarbonate level, electrolytes, A1c, pH, ketones, and serum glucose level. Data regarding insulin regimen and dosing, type of intravenous (IV) fluids, using of bicarbonate infusion, consciousness level, and feeding status were also collected.

The primary outcome of this study was to determine the difference in time to close the AG between patients with normal kidney function compared to those with CKD. A closed AG was defined as an AG of < 16 mmol/L. Secondary outcomes included hospital and intensive care unit (ICU) length of stay (LOS), and the incidence of hypoglycemic event during the first 24-hour. Hypoglycemia was defined as a blood glucose < 3.9 mmol/L. In addition, the effect of diabetes type, the insulin regimen, type of IV fluids, bicarbonate administration on time to close the anion gap were also evaluated.

Descriptive statistics were performed to summarize patient demographics and outcomes using mean ± standard deviation (SD) or median (interquartile range [IQR]) for the continuous variables and frequencies (percentages) for categorical variables, as appropriate. Student’s t-tests were used to assess the difference between the two groups in terms of time to close the anion gap. Sub-group analysis was done to determine the effect of diabetes type, insulin regimen, type of IV fluids, bicarbonate administration, and steroid administration on the time to close the anion gap between groups. Data were analyzed using SAS^®^ software, version 9.4 (SAS Institute Inc., Cary, NC).

## Results

Of 319 patients screened, 183 patients were included in the analysis. The normal kidney function group consisting of 79 patients (43.2%) and the CKD group comprising 104 patients (56.8%). Patients with renal function impairment were older compared to the control group (56.5 vs. 27.9) years. The majority of patients in the normal kidney function group had type I diabetes 66/79 (83.5%) while the majority of patients in the CKD group had type II diabetes 76/104 (73.1%). Most of the patients in the normal kidney function group had a diagnosis of DKA (94.9%) whereas the majority of patients with combined DKA/HHS event were in the CKD group (51/55; 92.7%). The average eGFR for patients with normal kidney function was 93.7 ± 32.5 mL/min/1.73m^2^ compared to 33.4 ± 14.3 mL/min/1.73m^2^ for patients with CKD. Decreased alertness was documented more in patients with CKD compared to patients with normal renal function (27.9% vs. 5.1%). Baseline demographics are presented in **Table 1**.

**Table 1:** Patients’ baseline characteristics.

Patients’ characteristics	Overall (n= 183)	Normal kidney function (n= 79)	Chronic kidney disease (n= 104)	*p*-value
Age in years	44.2 ± 24.3	27.9 ± 16.3	56.5 ± 22.2	**<0.0001**
BMI (kg/m^2^)	24.0 ± 5.7	23.0 ± 4.5	24.9 ± 6.3	**0.0197**
Gender				0.7394
Male	104 (56.8)	46 (58.2)	58 (55.8)	
Female	79 (43.2)	33 (41.8)	46 (44.2)	
Type of diabetes				**<0.0001**
Type 1	94 (51.4)	66 (83.5)	28 (26.9)	
Type 2	89 (48.6)	13 (16.5)	76 (73.1)	
Reason for admission				**<0.0001**
Isolated DKA	128 (69.9)	75 (94.9)	53 (51.0)	
Combined HHS/DKA	55 (30.1)	4 (5.1)	51 (49.0)	
Long-term diabetes management				**0.0004**
Insulin	115 (62.8)	60 (75.9)	55 (52.9)	
Insulin + oral anti-hyperglycemic agent	23 (12.6)	4 (5.1)	19 (18.3)	
Oral anti-hyperglycemic agents	11 (6.0)	0 (0.0)	11 (10.6)	
No documented therapy	34 (18.6)	15 (19.0)	19 (18.3)	
Consciousness level on admission				**0.0002**
Alert	149 (81.4)	75 (94.9)	74 (71.2)	
Decreased alertness	33 (18.0)	4 (5.1)	29 (27.9)	
Unconscious	1 (0.5)	0 (0.0)	1 (1.0)	
Chronic steroids use	9 (4.9)	0 (0.0)	9 (8.7)	**0.0073**
Presence of acute renal failure	53 (29.0)	2 (2.5)	51 (49.0)	**<0.0001**
Presence of acute liver failure	4 (2.2)	0 (0.0)	4 (3.8)	**0.0009**
Feeding Status				0.1905
PO	164 (89.6)	68 (86.1)	96 (92.3)	
NPO	18 (9.8)	11 (13.9)	7 (6.7)	
NGT	1 (0.5)	0 (0.0)	1 (1.0)	
Baseline laboratory values				
HbA1c (n=61)	12.2 ± 2.5	10.9 ± 1.8 (n=21)	12.8 ± 2.5 (n=40)	**0.0025**
Plasma glucose level (mmol/L)	33.1 ± 9.5	29.7 ± 9.2	35.7 ± 8.9	**<0.0001**
Scr (mmol/L)	162.7 ± 101.9	93.9 ± 27.2	214.9 ± 106.8	**<0.0001**
CrCl (ml/min)	55.5 ± 33.1	83.5 ± 27.2	34.2 ± 17.9	**<0.0001**
eGFR (mL/min/1.73m2)	57.0 ± 37.5	93.7 ± 32.5	33.4 ± 14.3	**<0.0001**
Anion gap (mmol/L)	27.7 ± 7.0	29.2 ± 6.2	26.5 ± 7.4	**0.0089**
Serum osmolality (mOsm/kg)	328.3 ± 28.1	317.9 ± 22.7	335.3 ± 29.3	**0.0001**
Sodium (mmol/L)	133.2 ± 8.5	133.7 ± 4.5	132.8 ± 10.6	0.4288
Potassium (mmol/L)	5.1 ± 0.8	4.9 ± 0.7	5.2 ± 0.9	**0.0422**
Magnesium (mmol/L)	0.9 ± 0.2	0.8 ± 0.2	1.0 ± 0.2	**<0.0001**
Phosphate (mmol/L)	1.6 ± 0.6	1.4 ± 0.5	1.7 ± 0.6	**0.0129**
Bicarbonate (mmol/L)	14.3 ± 6.8	12.9 ± 5.5	15.4 ± 7.6	**0.0119**
PH	7.2 ± 0.1	7.2 ± 0.1	7.2 ± 0.1	0.8978
Urine ketones level (n=116)	107.7 ± 50.1	127.5 ± 39.6	82.4 ± 51.0	**<0.0001**

Results are presented as frequency (%) or mean ± SD.

BMI, body mass index; CrCl, creatinine clearance; DKA, diabetic ketoacidosis; eGFR, estimated glomerular filtration rate; HHS, hyperosmolar hyperglycemic state; NGT, nasogastric tube; NPO, nothing by mouth; PO, oral; Scr, serum creatinine; SD, standard deviation. Bold values indicate statistical significant results.

The CKD group had higher plasma glucose level on admission compared with the normal renal function group (35.7 ± 8.9 vs 29.7 ± 9.2). In addition, patients with CKD presented with higher serum osmolality (335.3 ± 29.3 vs 317.9 ± 22.7), potassium (5.2 ± 0.9 vs 4.9 ± 0.7), phosphate (1.7 ± 0.6 vs 1.4 ± 0.5), but lower AG (26.5 ± 7.4 vs 29.2 ± 6.2) levels compared with patients with normal renal function (**Table 1**).

In terms of management, IV insulin bolus dose beside insulin infusion was given among 14 (17.7%) and 48 (46.2%) patients with normal and impaired kidney function, respectively. Both groups were similar with regard to the average bolus insulin dose, initial insulin infusion rate, and duration of continuous IV insulin infusion. However, a higher percentage of patients in the CKD group received IV bicarbonate infusion compared to patients in the normal renal function group (11.5% vs. 7.6%). **Table 2** presents details for the management strategies in both groups.

**Table 2:** Management strategies.

Patients’ characteristics	Overall (n= 183)	Normal kidney function (n= 79)	Chronic kidney disease (n= 104)	*p*-value
Administration of SubQ insulin on admission	24 (13.1)	8 (10.1)	16 (15.4)	0.1674
Insulin regimen				**<0.0001**
Continuous IV Infusion	114 (62.3)	64 (81.0)	50 (48.1)	
Continuous IV Infusion + Bolus	62 (33.9)	14 (17.7)	48 (46.2)	
Bolus	7 (3.8)	1 (1.3)	6 (5.8)	
Insulin Bolus Dose (units)	7.8 ± 2.4	7.6 ± 3.1	7.9 ± 2.3	0.7227
Initial Insulin Infusion Rate (u/hr)	5.8 ± 1.6	5.5 ± 1.4	5.9 ± 1.8	0.0954
Duration of Continuous IV Infusion (hr)	15.8 ± 11.1	15.3 ± 10.5	16.2 ± 11.6	0.5872
Type of IV fluid				0.1602
Sodium Chloride 0.9%	172 (94.0)	74 (93.7)	98 (94.2)	
Lactated Ringer's	5 (2.7)	4 (5.1)	1 (1.0)	
Other or not documented	6 (3.3)	0 (0.0)	6 (3.3)	
Use of Bicarbonate Infusion	18 (9.8)	6 (7.6)	12 (11.5)	0.3749

Results are presented as frequency (%) or mean ± SD.

IV, intravenous; SubQ, subcutaneous. Bold values indicate statistical significant results.

In terms of study outcomes, the time to close the AG was comparable between patients with CKD and patients with normal kidney function (**Table 3**). Among patients with CKD, the average time to close the AG was 24.5 ± 17.5 hours, while it was 22.6 ± 16.0 hours among patients with normal kidney function; and this difference was not statistically significant (*p*=0.4475). However, when considering patients with isolated DKA only, those with CKD had a slightly shorter time to close the AG compared to the other group, but this difference was not statistically significant (21.3 vs. 23.2 hours, *p*=0.503). Conversely, among patients with combined DKA/HHS, CKD patients took longer to close the AG compared to the normal kidney function group, but this result was not statistically significant (27.8 hours vs. 11.0 hours, *p*=0.113). Hospital and ICU LOS were both longer for patients with CKD compared to those with normal renal function, with duration of (5.2 ± 4.0 vs. 3.4 ± 2.5 days, *p*<0.001) and (4.0 ± 2.8 vs. 2.5 ± 1.8 days, *p*=0.045), respectively. The occurrence of hypoglycemic events was low in our study, with only four documented cases all among patients with CKD during the first 24 hours.

**Table 3:** Primary and secondary outcomes.

Patients’ characteristics	Overall (n= 183)	All patients			Isolated DKA			Combined DKA/HHS		
		NKF (n= 79)	CKD (n= 104)	*p*-value	NKF (n= 75)	CKD (n= 53)	*p*-value	NKF (n= 4)	CKD (n= 51)	*p*-value
Primary outcomes										
Mean time to close AG (hr)	23.6 ± 16.8	22.6 ± 16.0	24.5 ± 17.5	0.4475	23.2 ± 16.1	21.3 ± 13.5	0.5028	11.0 ± 7.9	27.8 ± 20.5	0.1128
Patients with AG closure within 12 hours	49 (26.8)	25 (31.6)	24 (23.1)	0.1948	23 (30.7)	10 (18.9)	0.1328	2 (50.5)	14 (27.6)	0.3390
Mean time to close AG (hr)	7.5 ± 3.2	7.8 ± 3.3	7.2 ± 3.2	0.5759	8.1 ± 3.2	9.3 ± 2.5	0.2873	4.5 ± 2.1	5.7 ± 2.8	0.5935
Secondary outcomes										
Hospital length of stay (days)	4.4 ± 3.6	3.4 ± 2.5	5.2 ± 4.0	**0.0004**	3.0 ± 1.7	3.9 ± 3.0	0.0714	10.0 ± 5.0	6.7 ± 4.5	0.1611
ICU admission	45 (24.6)	19 (24.1)	26 (25.0)	0.8826	19 (25.3)	19 (35.9)	0.1996	0 (0.0)	7 (13.7)	0.4277
ICU length of stay (days)	3.4 ± 2.5	2.5 ± 1.8	4.0 ± 2.8	**0.0453**	2.5 ± 1.8	3.5 ± 2.4	0.1366	--	5.3 ± 3.6	--
Hypoglycemic events within 24 hours	4 (2.2)	0 (0.0)	4 (3.9)	0.1349	0 (0.0)	4 (7.5)	0.0274	0 (0.0)	0 (0.0)	--

Results are presented as frequency (%) or mean ± SD.

AG, Anion Gap; CKD, Chronic kidney disease; DKA, diabetic ketoacidosis; HHS, hyperosmolar hyperglycemic state; ICU, intensive care unit; NKF, Normal kidney function. Bold values indicate statistical significant results.

In the sub-group analysis, there were not significant difference in the time to close the AG among patients with type 1 or type 2 diabetes, patients who received IV bolus insulin dose versus continuous IV infusion only, patients who received normal saline or lactated ringers, and in patient administered sodium bicarbonate in both groups (**Table 4**). However, subcutaneous insulin dose was given on admission among 8 (10.1%) and 16 (15.4%) patients with normal and impaired kidney function, respectively. Time to close the AG was significantly shorter among patients with normal kidney function who received an insulin subcutaneous dose with a mean of 11.2 ± 10.2 hours compared to 26.9 ± 19.3 hours for patients with CKD (MD 15.7 ± 16.8 hours, *p*=0.045).

**Table 4:** Sub-group analysis for mean time to close anion gap (hours).

Outcome	Normal kidney function (n= 79)	Chronic kidney disease (n= 104)	Mean Difference ± SD	*p*-value^*^
Mean time to close anion gap (hr), mean (SD)	22.6 ± 16.0	24.5 ± 17.5	1.9 ± 16.9	0.447
Type of diabetes**				
Type 1 DM, (All, n= 94)	23.7 ± 15.9	20.6 ± 14.1	3.1 ± 15.4	0.382
Type 2 DM, (All, n= 89)	16.9 ± 15.7	25.9 ± 18.5	9.0 ± 18.1	0.102
• Type 2 DM (isolated DKA, n=34)	19.6 ± 17.9	22.2 ± 13.0	2.6 ± 14.3	0.641
• Type 2 DM (Combined DKA/HHS, n=55)	11.0 ± 7.9	27.8 ± 20.5	16.8 ± 20.0	0.113
Insulin regimen				
Continuous IV Infusion	23.2 ± 16.5	24.0 ± 17.9	0.8 ± 17.1	0.803
Continuous IV Infusion + Bolus	21.1 ± 13.7	25.1 ± 16.8	4.1 ± 16.2	0.412
Bolus	3.0 ± 0.0	22.9 ± 23.3	19.9 ± 23.2	0.478
Chronic steroids use				
No	22.5 ± 16.0	24.5 ± 17.8	2.0 ± 16.9	0.451
Yes				
Reason for admission				
Isolated DKA	23.2 ± 16.1	21.3 ± 13.5	1.8 ± 15.1	0.503
Combined DKA/HHS	11.0 ± 7.9	27.8 ± 20.5	16.8 ± 20.0	0.113
Type of IV fluid				
Sodium Chloride 0.9%	23.1 ± 16.2	24.7 ± 17.4	1.6 ± 16.9	0.532
Lactated Ringer's	17.1 ± 8.9	16.0 ± 0.0	1.1 ± 8.9	0.917
Other or not documented	2.0 ± 0.0	11.0 ± 0.0	–	–
Administration of SubQ insulin on admission				
No	23.8 ± 16.1	24.2 ± 17.2	0.4 ± 16.7	0.888
Yes	11.2 ± 10.2	26.9 ± 19.3	15.7 ± 16.8	**0.045**
Use of bicarbonate infusion				
No	22.2 ± 15.9	24.4 ± 17.5	2.2 ± 16.8	0.398
Yes	26.7 ± 18.4	24.6 ± 17.7	2.1 ± 17.9	0.820

*p-value is from the t-test for the subgroup analysis.

**All patients with Type 1 DM had isolated DKA (n=94), while patients with Type 2 DM had isolated DKA (n=34) or Combined DKA/HHS (n=55).

DKA, diabetic ketoacidosis; HHS, hyperosmolar hyperglycemic state; IV, intravenous; SubQ, subcutaneous; SD, standard deviation. Bold values indicate statistical significant results.

## Discussion

The findings of our study shed light on the management strategies and outcomes of DKA and combined DKA/HHS in patients with normal kidney function and those with CKD. Our analysis included 183 patients, of which 104 patients in the CKD group. In our study, the time to close the AG was comparable between patients with CKD and patients with normal kidney function with a non-significantly longer time needed for the management of patients with CKD.

Firstly, we observed that patients with CKD were older compared to patients in the normal kidney function group, which is consistent with previous research highlighting the association between CKD and aging (16). Furthermore, the majority of patients in the normal kidney function group had a diagnosis of DKA, while the majority of the combined DKA/HHS cases were in the CKD group. This can be explained by the fact that most of the patients in the normal kidney function group had type I diabetes whereas DKA is a known complication in this type of diabetes, while in the CKD group the majority of patients had type II diabetes, therefore more cases of combined DKA/HHS were reported in this group. In addition, the results showed that patients with CKD presented with higher blood glucose, serum osmolality, and potassium levels on admission compared with the normal kidney function group. These findings align with a previous retrospective study that evaluated the clinical characteristics and outcomes of patients with DKA and end stage renal disease (ESRD) on hemodialysis (HD) (17). However, they reported higher AG level in the ESRD group which could be explained by including only patients with isolated DKA in their study.

Our study also assessed the time required to close the AG. Interestingly, we found that the time to close the AG was similar between patients with CKD and those with normal kidney function. However, in patients with isolated DKA, those with CKD tended to have a shorter time to close the AG, although this difference was not statistically significant in our study. It is not clear why but it can be related to a slower rate in the insulin clearance in those patients. Galindo et al. found that the time for hyperglycemia correction to less than 250 mg/dL was longer in patients with DKA and ESRD compared with patients with preserved renal function (8.4 ± 2.6 vs. 7.2 ± 3.1 hours, *p*=0.03) (17). However, in their study the rate of glucose reduction in the first 24 hours was significantly higher in patients with ESRD (-596.1 ± 315.2 vs. -268.0 ± 154.3 mg/dL, *p*<0.001). The time to close the AG or DKA resolution was not evaluated in their study. Several studies recommend insulin infusion rate reduction and/or avoiding the bolus dose in patients with DKA and ESRD on HD to prevent rapid reduction in blood glucose level and rapid changes in osmolality (17–19). The recommendation is to decrease the insulin infusion rate to 0.05-0.07 unit/kg/hr.

In patients with combined DKA/HHS, the time to close the AG was longer in the CKD group, although this difference was not statistically significant. This may be related to the presence of more mixed acidosis scenarios as observed by the sodium bicarbonate utilization in this group. These findings suggest that the presence of CKD may influence the resolution time of the AG in specific DKA presentations. Importantly, patients with CKD experienced longer hospital stays and ICU stays compared to those with normal renal function. This indicates that CKD poses additional challenges in the management of DKA, potentially due to the complexity of medical comorbidities associated with renal impairment.

Hypoglycemic events were infrequent in our study, with only a small number of CKD patients experiencing documented hypoglycemia within the first 24 hours. This suggests that the risk of hypoglycemia during DKA treatment may not be significantly increased in patients with CKD. Of notice, the study by Galindo et al. reported more frequent hypoglycemic events compared to our study, and these events were significantly higher in the ESRD group (17). This difference in the occurrence of hypoglycemia compared to our results could be related to the differences in the study populations, as Galindo et al. included only ESRD patients, as well as differences in the timeframe for evaluating this endpoint, since our study assessed hypoglycemic events only during the first 24 hours.

Intravenous fluid resuscitation is a cornerstone therapy in the management of DKA and HHS. Our results showed that the majority of patients in both groups received normal saline as part of their treatment; however, the specific infusion rate of IV fluids was not collected in this study. The current clinical guidelines recommend starting with isotonic fluid at a rate of 15-20 mL/kg/hour or 1-1.5 L in the first hour, followed by a subsequent rate of 250-500 mL/hour (2). However, for patients with cardiac or renal compromise, close monitoring of serum osmolality, renal and cardiac function, and mental status during fluid replacement is necessary to avoid volume overload and further complications (2). Previous studies have reported higher rates of hospital complications like volume overload, pulmonary edema, and the need for mechanical ventilation in patients with DKA and ESRD compared to those with preserved renal function (17). In patients with DKA and ESRD, the lack of osmotic diuresis due to hyperglycemia can lead to expansion of the extracellular fluid volume and overload. Previous studies have recommended a more cautious approach, using 250-500 mL fluid boluses guided by the patient's volume status, and considering HD in cases of severe volume overload (18, 19). However, the appropriate fluid management approach has not been well studied in patients with other CKD stages who are not on maintenance HD.

Subgroup analyses were conducted to explore potential differences in various clinical factors. Since the majority of patients in the normal kidney function group had type 1 diabetes while the majority of CKD patients had type 2 diabetes, we analyzed the primary outcome based on the type of diabetes. The results of the subgroup analysis showed no significant difference in the average time to close the AG between the two groups for both type 1 and type 2 diabetes. However, we noted that in type 1 diabetes, patients with CKD had a shorter time to close the AG compared to patients with normal renal function, although this difference was not significant. This may be due to an increased insulin effect resulting from delayed insulin clearance. Conversely, in type 2 diabetes, patients with CKD showed a non-significantly longer time to close the AG compared to those with normal kidney function, which could be attributed to insulin resistance. Furthermore, we found that patients with normal kidney function who received a subcutaneous insulin dose upon admission had a significantly shorter time to close the AG compared to those with impaired kidney function. This highlights the potential benefit of early subcutaneous insulin administration in patients without renal impairment.

Our study has several limitations. Firstly, it was conducted at a single center, which may limit the generalizability of the findings. Additionally, the sample size was relatively small, particularly in certain subgroups, which may have limited the statistical power to detect significant differences. We included a mixed group of DKA and HHS cases, however, due to the study design this will be an inherent dilemma. Furthermore, we did not evaluate the differences in the infusion rates of IV fluid and electrolytes replacement between the two groups which is a critical component of DKA management. Also, hospital complications like volume overload, electrolytes disturbances, and the need for mechanical ventilation were not assessed in this study.

In addition to the limitations mentioned in our study, there are several advantages worth noting. Firstly, our study contributes to the existing literature by specifically focusing on the management and outcomes of DKA in patients with CKD not on HD. This allows for a better understanding of the unique challenges and considerations in this patient population. Secondly, our study explored important clinical factors associated with DKA management, such as the time to close the AG and the occurrence of hypoglycemic events, providing valuable insights for patient care. Despite these advantages, future research should address the limitations through larger, multicenter studies to validate and strengthen the evidence in this field.

## Conclusion

Our study provides valuable insights into the management and outcomes of DKA in patients with normal kidney function and CKD. In this study, the time to close the AG was similar between patients with impaired kidney function and patients with normal kidney function. Further research involving larger multicenter studies is warranted to validate these findings and explore additional factors that may impact DKA management in patients with CKD.

## Data Availability

The raw data supporting the conclusions of this article will be made available by the authors, without undue reservation.
